# Role of AmpC-Inducing Genes in Modulating Other Serine Beta-Lactamases in *Escherichia coli*

**DOI:** 10.3390/antibiotics11010067

**Published:** 2022-01-06

**Authors:** Dhriti Mallik, Diamond Jain, Sanjib Bhakta, Anindya Sundar Ghosh

**Affiliations:** 1 Department of Biotechnology, Indian Institute of Technology Kharagpur, Kharagpur 721302, India; dhritims@gmail.com (D.M.); diamondjain@iitkgp.ac.in (D.J.); 2 Department of Biological Sciences, Institute of Structural and Molecular Biology, Birkbeck, University of London, Malet Street, London WC1E 7HX, UK; s.bhakta@bbk.ac.uk

**Keywords:** beta-lactamase, antimicrobial resistance, peptidoglycan recycling, therapeutic target, WHO-priority pathogen, *E. coli*

## Abstract

The consistently mutating bacterial genotypes appear to have accelerated the global challenge with antimicrobial resistance (AMR); it is therefore timely to investigate certain less-explored fields of targeting AMR mechanisms in bacterial pathogens. One of such areas is beta-lactamase (BLA) induction that can provide us with a collection of prospective therapeutic targets. The key genes (*ampD*, *ampE* and *ampG*) to which the AmpC induction mechanism is linked are also involved in regulating the production of fragmented muropeptides generated during cell-wall peptidoglycan recycling. Although the involvement of these genes in inducing class C BLAs is apparent, their effect on serine beta-lactamase (serine-BLA) induction is little known. Here, by using ∆*ampD* and ∆*ampE* mutants of *E. coli*, we attempted to elucidate the effects of *ampD* and *ampE* on the expression of serine-BLAs originating from *Enterobacteriaceae*, *viz*., CTX-M-15, TEM-1 and OXA-2. Results show that cefotaxime is the preferred inducer for CTX-M-15 and amoxicillin for TEM-1, whereas oxacillin for OXA-2. Surprisingly, exogenous BLA expressions are elevated in ∆*ampD* and ∆*ampE* mutants but do not always alter their beta-lactam susceptibility. Moreover, the beta-lactam resistance is increased upon *in trans* expression of *ampD*, whereas the same is decreased upon *ampE* expression, indicating a differential effect of *ampD* and *ampE* overexpression. In a nutshell, depending on the BLA, AmpD amidase moderately facilitates a varying level of serine-BLA expression whereas AmpE transporter acts likely as a negative regulator of serine-BLA.

## 1. Introduction

The unique features of the bacterial cell wall make it a promising target for the majority of antimicrobial agents [1,2]. The presence of peptidoglycan (PG) layer in the cell wall helps bacteria to maintain their rigidity, cell shape and also protects them from environmental upheavals [3]. The biosynthesis and recycling of cell-wall PG involve multiple enzymatic steps in various locations of the bacterial cells, which are widely investigated for developing antimicrobial agents [1,2,4,5]. PG is composed of polymeric chains containing repeating units of *N*-acetylglucosamine (NAG) and *N*-acetylmuramic acid (NAM) to which pentapeptide moieties are linked. Next, the adjacent muramoyl pentapeptides are cross-linked at the fourth D-alanine of one chain with *meso*-diaminopimelic (*m*-DAP) acid at the third position of the other chain [6,7]. The enzymatic steps in cross-link formation are catalyzed by a set of ectoproteins (tethered at the outer facet of cytoplasmic membrane) present in the periplasm known as penicillin-binding proteins (PBPs) [8,9]. Beta-lactam antibiotics target these PBPs to stall the final metabolic steps of PG biogenesis and till today, it is the drug of choice to tackle bacterial infections due to the selective toxicity [10]. However, the constant evolution of beta-lactam hydrolyzing enzymes, beta-lactamases (BLA), is limiting their use in clinical settings. These BLAs are broadly classified into four classes (A–D) by Ambler et al. [11]. The enzymes from classes A, C and D require an active-site serine residue for their activity whereas class B are metallo-BLAs that require zinc ions to exert their effect on beta-lactams [11].

The expression of BLAs in presence of beta-lactams has been correlated with PG recycling in Gram-negative bacteria [12]. The recycling pathway of the cell wall is precisely regulated along with its synthesis to avoid cell lysis and preserve cell-wall integrity [5]. The activity of the lytic transglycosylases upon the NAG-NAM glycosidic (β-D 1-4) linkage of murein generates PG fragments. The action of this group of enzymes may either be exolytic or endolytic, generating a multitude of different fragments [13]. Although there are certain discrepancies in the actions of some proteins involved in the cellular uptake of PG, apparently the cytoplasmic membrane-bound AmpG permease is found responsible for the transport of the muropeptides released from the PG in periplasm into the cytoplasm. Strains lacking *ampG* liberate approximately 40% of the PG fragments to the host environment [14]. Once the fragments reach the cytosol, they are acted upon by the AmpD amidase that separates the disaccharide GlcNAc–anhMurNAc from the peptide segment of PG fragment, following which the disaccharide and the peptide fragments are processed separately for the de novo synthesis of PG [15]. It is reported that the attack of beta-lactams leads to the generation of a larger amount of muropeptides in periplasm, thereby breaking the balance of PG synthesis and recycling [16].

To date, apart from AmpC, the regulation of BLA belonging to any other class has not been established except for the one recently reported where induction of DHA-1 β-lactamase is shown similar to that of chromosomal AmpC β-lactamase, but the actual mechanism is not investigated [17]. The AmpC BLA is a member of Ambler class C but there are two other classes of serine BLA, *viz*., class A and class D. However, except the spectra of hydrolysis [18] and deduced structures of BLA categorized as class A and D, little is known about the genes that affect their expression. In this study, we have aimed to understand whether genes involved in the expression of *ampC* in Gram-negative bacteria can modulate the expression of other serine BLAs in *E. coli.* The host organism of choice was *E. coli* as all the BLA genes used in this study were isolated from the clinical strains of *E. coli*. It is one of the most rapidly evolving multi-drug resistant (MDR) bacteria for which we do not have a specific counteracting drug so far. It is speculated that each beta-lactam antibiotic acts as an inducer for different sets of BLA [19], where some can induce the expression of a specific BLA while others do not. Here, we have intended to experimentally correlate such variations in the expression of clinically predominant Class A (CTX-M and TEM type) and Class D (OXA) BLAs in presence of sub-inhibitory concentrations of respective beta-lactams in the presence or absence of the genes involved in AmpC induction and cell-wall PG recycling. Though not so far found in *E. coli*, *ampR* regulator helps in the process of induction of AmpC in some species of *Enterobacteriaceae* and other bacteria [14,15,16]. This has led us to investigate the existence of any secondary regulator of AmpC induction present in *E. coli* that may substitute AmpR. In addition, although the members of the peptidoglycan recycling pathway are known, the functions of *ampD* and *ampE* in the process of AmpC induction are not elucidated. Here, we have addressed the same to find the correlation of AmpD and AmpE in the AmpC induction process.

## 2. Results and Discussion

### 2.1. Deletion of AmpC Induction Genes Altered the Beta-Lactam Sensitivity of E. coli

To check whether the absence of *ampC* induction pathway genes *ampD* and *ampE* have any effect on the beta-lactam susceptibility, the same was checked for *ampD* and *ampE* deletion mutants using the standard drug susceptibility testing (DST) method. The Δ*ampD* strain exhibited a 2-fold enhancement in susceptibility to amoxicillin and cefalothin as compared to the parent strain (Table 1). Similarly, the Δ*ampE* strain showed a 2-fold increased sensitivity to amoxicillin, cefalothin and cefoxitin. In line with this observation, the effect of elevation of the beta-lactam susceptibility was also reported in the case of the Δ*ampG* mutant [20].

AmpC is a chromosomal BLA, commonly present in most Gram-negative bacteria, and is constitutively expressed in *E. coli* [21,22]. To nullify the effect of this BLA, drug susceptibility testing (DST) of the mutants were also conducted in an *ampC* deficient background (Table 1). The double deletion mutants (Δ*ampDC* and Δ*ampGC*) did not show an increase in sensitivity as compared to single deletion mutants. In contrast, Δ*ampCE* double deletion mutants decreased the susceptibility to the selected beta-lactams used.

The enhanced susceptibility of single deletion mutants to the beta-lactam antibiotics might indicate the probable role of *ampD* and *ampE* in maintaining the intrinsic beta-lactam resistance of *E. coli* cells. Similar observations have been reported in *Citrobacter freundii*, where an individual absence of *ampD* and *ampE* increases the susceptibility to the beta-lactams that are tested against the bacterial strain [23]. The role of AmpE transporter has not been established, though is predicted to be involved as an indirect beta-lactam sensor that may trigger a bacterial defense system or PG remodeling. Its absence might increase bacterial susceptibility to beta-lactams as these antibiotics target PG synthesizing enzymes, i.e., PBPs. As AmpD amidase is involved in the processing of PG fragments during PG remodeling [24], its absence could have led to the increased vulnerability of PG during beta-lactam attack, leading to increased susceptibility to the antibiotics.

As the double deletion mutant Δ*ampDC* did not show increased sensitivity, we interpret that constitutive *ampC* expression does not contribute much to the resistance of *E. coli* against the beta-lactams. This suggests that the role of AmpD in maintaining the intrinsic resistance of *E. coli* is likely to be independent of AmpC BLA.

### 2.2. Each Serine Beta-Lactamase Displayed Its Characteristic Spectrum of Beta-Lactam Hydrolysis

To reconfirm the activity of serine-BLA on various beta-lactams, the serine-BLAs under study were cloned and expressed in *E. coli* 25113, and their hydrolysis spectra were determined by checking their resistance to a range of beta-lactam antibiotics. Depending on the type of serine-BLA, variations in the resistance against characteristic antibiotics were observed (see Table 2). CTX-M-15 is an ESBL that shares the namesake for its cefotaxime hydrolyzing ability [25]. We noted a 32-fold increment of cefotaxime resistance in *E. coli* harboring CTX-M-15 as well as a significant rise in the resistance against other beta-lactams tested. On the other hand, the hydrolysis spectra of TEM-1 encompass the penicillin group, and we observed an 8-fold increased resistance against amoxicillin, whereas an Ambler class D enzyme, OXA-2, imparted a 4-fold resistance to oxacillin and a 16-fold increase of resistance against both amoxicillin and ampicillin [11]. Therefore, based on the above results, we infer that the decrease in beta-lactam sensitivity might act as a piece of indirect evidence for the expression of cloned BLAs.

### 2.3. Induction of Classes A and D Beta-Lactamases in E. coli by Sub-Inhibitory Concentrations of Beta-Lactam Antibiotics

To explore the mechanism of induction of class A and class D serine-BLA, expression of the same in presence of beta-lactams was studied. Initially, the sub-inhibitory concentrations of the beta-lactams were determined and 1/8th of the minimum inhibitory concentrations (MICs) were fixed as the inducing dose for BLAs in *E. coli* 25113. The BLAs were expressed using the exogenous pBAD promoter system without any inducer for the particular promoter. The strains were grown in presence of beta-lactams as an inducer for BLAs and the cell lysates were used to elucidate BLA by assessing the hydrolysis of the chromogenic cephalosporin, nitrocefin (see Section 3 for details)*.*

**Figure 1:**
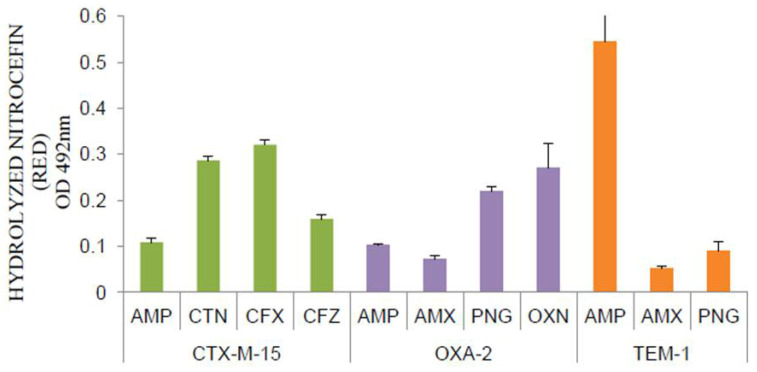
Induction of serine beta-lactamases—CTX-M-15, OXA-2 and TEM-1 by beta-lactam antibiotics in *E. coli* 25113. Antibiotics: AMP—ampicillin, CTN—cefalothin, CFX—cefotaxime, CFZ—ceftazidime, AMX—amoxicillin, PNG—penicillin G and OXN—oxacillin.

Varying levels of BLA expressions were observed for each BLA, depending upon the beta-lactam employed as an inducer (Figure 1). CTX-M-15 level was higher when cefalothin and cefotaxime were used as inducers, showing ~2.6- and ~3-fold increased expression, respectively, as compared to ampicillin. Ceftazidime induction was marginally better than ampicillin (~1.5-fold). The expression of OXA-2 was comparatively higher than amoxicillin by ~1.4-, ~3- and ~3.8-fold when induced by ampicillin, penicillin G and oxacillin, respectively, indicating that oxacillin was the best inducer of OXA-2 followed by penicillin G. Expression of TEM-1 was highest in the presence of ampicillin used as an inducer with an approximate 11-fold higher level of expression than that of amoxicillin and 6-fold higher than that of penicillin G. The use of nitrocefin as a secondary (reporter) substrate for BLA expression in presence of beta-lactams, employed as the inducers, confirmed the activity of the enzyme, which might be due to the higher expression of enzymes than the higher catalytic efficiency of the enzymes against certain antibiotics. As a result, it is noticed that serine-BLA expression varies in response to different beta-lactams. Such responses are reported in *Enterobacter cloacae* concerning the class I cephalosporinases [26]. Therefore, serine-BLAs other than the members of class C BLAs also differ in their induction levels in response to different beta-lactam antibiotics employed for induction. We speculate that there could be a possibility of the existence of a stable second messenger system such as BlaR from *Bacillus licheniformis* [27,28,29], which might be involved in modulating the expression of all the cloned BLAs relying on their leaky expression from pBAD promoter (as arabinose was never used for expression). Although the mode of induction by the beta-lactams affecting the expression of beta-lactamases from an extraneous promoter needs further study, the inducibility of the arabinose promoter is established in the expression system with the coexistence of the other promoter system, such as *trc* promoter or even *araB*, which is shown to have regulations on the co-expression of *Bacillus* genes [30]. Moreover, it is reported earlier that the arabinose inducibility is host-dependent and often could not be attributed to the strains with a mutation in the *ara* operon [30]. Given that the *ara* operon of the host is intact as in our case, there may be a possibility of the existence of a secondary transducer to control the *in trans* expression from *araB* promoter present in the plasmid.

### 2.4. AmpD Expression Leads to the Alterations in Beta-Lactamase Expression

AmpD amidase processes the 1,6 anhydro-muropeptides generated by the action of NagZ on peptidoglycan catabolites [31], which are redirected to PG remodeling after the addition of UDP. The attack of beta-lactams leads to the generation of extra muropeptides, which are not processed by AmpD amidase and replace the UDP-pentapeptides to convert the AmpR regulator into a transcriptional activator conformation, thereby initiating the expression of AmpC BLA [32]. Thus, AmpD amidase acts as a negative regulator of AmpC expression. The unavailability of the AmpD amidase in the Δ*ampD* mutants might increase AmpC expression and also the beta-lactam resistance (Table S2). However, *E. coli* lacks AmpR regulator and so the constitutively expressing *ampC* does not enhance the intrinsic resistance of *E. coli* in absence of *ampD* [33]. Furthermore, the absence of *ampD* and the consequent absence of the processed muropeptides might reduce peptidoglycan integrity, which may be the reason for the increased susceptibility of *E. coli* in presence of beta-lactams. To confirm this hypothesis, we ectopically expressed AmpD amidase in *E. coli* Δ*ampD*, which efficiently complemented its activity leading to the decrease in the susceptibility as compared to the *E. coli* Δ*ampD* strain (Table 3). Seeing as the presence of AmpD amidase assists in maintaining the intrinsic resistance of *E. coli*, the generated fragments of PG, either in the absence or in presence of *ampD*, may also help in redirecting the pathway either towards peptidoglycan recycling or to BLA induction.

Therefore, to understand the role of AmpD amidase in serine-BLA expression, three BLAs were cloned from clinical isolates, expressed in host cells, both in the absence and presence of *ampD*, and their beta-lactam susceptibilities were determined. Simultaneous *in trans* overexpression of both AmpD amidase and CTX-M-15 or OXA-2 enhanced the resistance of the harboring cell as compared to that of wild type *E. coli* (Table 4).

In the case of class A enzyme TEM-1, overexpression of AmpD amidase resulted in the enhancement of beta-lactam sensitivity in presence of BLA. This is in line with the explanation that the amidase processes the peptidoglycan fragments for peptidoglycan remodeling. The peptide fragments generated, after the action of AmpD amidase, act as molecular signals to decide the direction of progression of the pathway, which can either be peptidoglycan recycling or BLA induction. If there is an accumulation of the muropeptides in the cytoplasm, it leads to the de-repression of BLA expression as a counteraction to the rapid peptidoglycan breakdown mostly mediated by a beta-lactam attack.

#### Variability Exists in the Expression of Beta-Lactamases in Δ*ampD* Mutant

**Figure 2:**
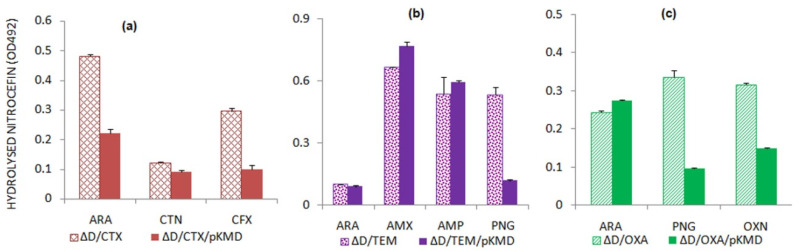
Relative beta-lactamase induction by arabinose and characteristic substrates of the beta-lactamases abbreviated as (**a**) CTX (CTX-M-15); (**b**) TEM (TEM-1); and (**c**) OXA (OXA- 2) in Δ*ampD* mutated (ΔD) and Δ*ampD* complemented strains (ΔD/pKMD) as measured by the nitrocefin hydrolyzing assay. Inducers used: ARA—arabinose; CTN—cefalothin; CFX—cefotaxime; AMX—amoxicillin; AMP—ampicillin; PNG—penicillin G; OXN—oxacillin.

The effect of AmpD amidase on the induction of BLA expression was checked by expressing each BLA in *ampD* deficient and *ampD* sufficient *E. coli* cells. Beta-lactams were used as inducers for the BLA expression and assayed by nitrocefin hydrolysis using the lysates of the cells expressing BLAs (Figure 2).

We observed a variable effect of AmpD amidase on the expression of each BLA in line with the antibiotic sensitivity assay. In most cases, the absence of *ampD* resulted in the higher expression of BLAs. However, in the case of TEM-1, expression levels were similar, both in the absence and presence of *ampD*. The AmpD amidase is responsible for preparing the PG fragments for the addition of the UDP moiety; the step commits them to be directed into the murein turnover pathway as opposed to the BLA induction pathway. In the absence of AmpD amidase, the accumulation of these fragments functions as a trigger to induce the expression of BLAs [34].

The induction of BLAs do not require the direct penetration of beta-lactams into the cell, therefore a signaling molecule must initiate the transcription of the BLA gene within the cytoplasm [35]. It is reported that AmpD amidase is likely to be involved as a mediator for the expression of BLAs.

### 2.5. AmpE Acts as a Negative Regulator of Beta-Lactamase Expression

The gene *ampE* encodes a transporter which is proposed to act as a sensor in the induction of BLAs due to the presence of the S-X-X-K motif of PBPs, although it does not bind to beta-lactams as the KTG motif, required for the activity, is absent in AmpE [19]. Moreover, it is suggested that AmpE might enhance the activity of AmpD in *E. coli* [19]. The absence of the AmpE transporter resulted in a 2-fold increase in amoxicillin susceptibility of *E. coli*, denoting its participation in BLA expression (Table S3). We observed an increase in the beta-lactam sensitivity upon expression of BLA in presence of AmpE transporter as compared to the expression of BLA in the Δ*ampE* mutant (Table 5). Concerning CTX-M-15, there was a 2-fold increase in ampicillin sensitivity and a 4-fold increase in cefotaxime sensitivity in presence of *ampE* expressed *in trans*. The most pronounced effect was noted in the case of TEM-1 expressed in presence of AmpE transporter. The increase in the susceptibility of Δ*ampE*/pKME/TEM-1 to amoxicillin and ampicillin were 32-fold and 64-fold, respectively, compared to the *ampE* mutant harboring the same BLA. Effect of expression of *ampE* transporter in presence of OXA-2 exhibited effects which were comparable to CTX-M-15. Increased beta-lactam sensitivity upon *in trans* expression of AmpE transporter in most cases may probably be due to the increased accumulation of transporter proteins that in turn helps in altering the permeability within the cell concerning the inflow and outflow of the substances from the bacterial cell. Beta-lactams mimic the D-Ala-D-Ala of the pentapeptide chain due to which the PG synthesizing enzymes are unable to form the cross-linked peptidoglycan layer. Therefore, AmpE transporter expression in an AmpD amidase-sufficient cell might allow the transport of a copious amount of beta-lactams along with the fragments that are destined for recycling. This could be a reason for the reduced resistance in the cells expressing both BLA and increased levels of AmpE transporter.

#### Presence of AmpE Effect on the Induction of Serine Beta-Lactamases by Beta-Lactams

**Figure 3:**
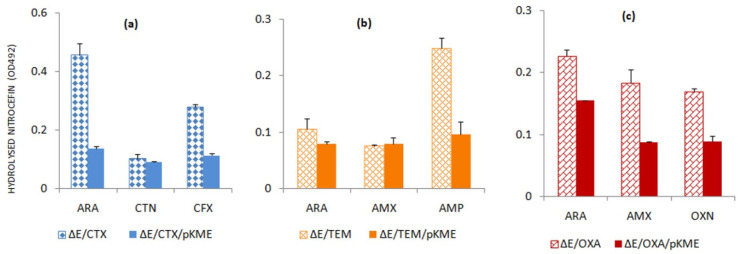
Differential induction by arabinose and characteristic substrates of the beta-lactamases abbreviated as (**a**) CTX (CTX-M-15); (**b**) TEM (TEM-1); and (**c**) OXA (OXA-2) in Δ*ampE* mutated (ΔE) and Δ*ampE* complemented strains (ΔE/pKME) as measured by nitrocefin hydrolyzing assay. Inducers used: ARA—arabinose; CTN—cefalothin; CFX—cefotaxime; AMX—amoxicillin; AMP—ampicillin; and OXN—oxacillin.

Change in beta-lactam sensitivity observed concerning the AmpE transporter indicates the possible role of its gene in the maintenance of beta-lactam resistance. Differential behavior was observed in the case of each BLA that led us to study the inducibility of these BLAs in absence and presence of *ampE*. As evident from Figure 3, the expressions of all three serine-BLAs tested are significantly higher in the absence of the gene of AmpE transporter whether it was induced by arabinose or by beta-lactam antibiotics. These results comply with the obtained beta-lactam sensitivity values (Table 5). Therefore, it can be speculated that the AmpE transporter might act as a negative regulator of BLA expression akin to the elucidated role of the AmpD amidase.

The generation of muropeptides leads to the induction of expression of chromosomal *ampC* (a class C BLA) [12]. Upon activity of beta-lactams, possibly at the sub-inhibitory level, the PG fragments are transported back to cytoplasm with the assistance of AmpG permease. Recently, it has also been reported that suppression of AmpG permease can efficiently inhibit both BLA induction and biofilm formation [20]. The accumulated cytoplasmic PG fragments help in the triggering of the AmpC induction via a transcriptional regulator, AmpR (a lysR type of regulator) [15,16]. The *ampR* regulator is repressed by the binding of cell-wall synthesis precursor UDP-MurNAc-pentapeptide, which are displaced by the anhydro-MurNAc-oligopeptides during beta-lactam attack leading to the expression of AmpC beta-lactamase [12]. The *ampR* gene is placed upstream to *ampC* in the genomes of *Enterobacteriaceae* such as *Citrobacter*, *Enterobacter* and *Serratia* species, though it is absent in *E. coli*, which has a low constitutive level of *ampC* expression [14,36]. The *blaA* gene of *Shewanella oneidensis* is induced in the presence of ampicillin akin to *ampC*, though it lacks *ampR* homologue for its regulation, suggesting a likelihood of the existence of an uncommon regulatory mechanism [37]. Thus, there is a possibility that *E. coli* may regulate a branch point without AmpR. As per the report, AmpD amidase possibly acts as a negative regulator that dissociates stem peptides from the anhydro-MurNAc or GlcNAc–anhydro-MurNAc, leading to the reduction in the concentration of AmpC-inducing peptides [24]. Though it is too early to provide constructive opinion on the effects of AmpD and AmpE on serine beta-lactamase expression, here we indicate that AmpD amidase may act as a moderate facilitator of serine beta-lactamase expression at a varying level, whereas the role of AmpE transporter might be a negative regulator of the expression of BLA.

## 3. Materials and Methods

### 3.1. Strains, Plasmids and Chemicals

*Escherichia coli* (CGSC-BW25113) was used as a host strain for genetic modifications. *E. coli* XL1-Blue was used as a maintenance host for recombinant plasmids. The recombinase gene cloned under an arabinose-inducible expression system in temperature-sensitive plasmid pKD46 with an ampicillin resistance marker that was used for genetic manipulations. The arabinose-inducible expression vectors pBAD18cam and pBAD18Kan were used for cloning and subsequent expression. The recombinant plasmids and genetically manipulated *E. coli* strains are mentioned in Table 3. The BLAs CTX-M-15, TEM-1 and OXA-2 were separately cloned in pBAD18-Cam as part of a previous study. The BLAs were originally isolated from clinical strains of *E. coli*. Unless otherwise specified, all bacterial cultures were grown and maintained in Luria–Bertani broth at 37 °C and Mueller–Hinton broth medium was used for drug susceptibility testing. Enzymes and antibiotics used in the study were procured from New England Biolabs (Beverly, MA, USA) and Sigma Chemical Company (St. Louis, MO, USA), respectively. Nitrocefin was acquired from Calbiochem (Merck, Germany).

### 3.2. Genetic Manipulations in E. coli BW25113

The strategy for genetic manipulations followed here was devised by Datsenko and Wanner (2000) [38] with certain modifications as adopted from Mallik et al. (2018) [20]. All the strains and plasmids used/created in the study are given in Table 3. *E. coli* BW25113 was transformed with temperature-sensitive plasmid pKD46 (marker: ampicillin 100 μg/mL; growth temperature: 30 °C) that carries the recombinase gene induced by arabinose. The expression was induced with 0.2% arabinose, and competent cells were prepared. The kanamycin cassette (kanr) was amplified along with FRT (flippase recognition target) sites on either side such that the amplicon is flanked on both sides by portions of sequences adjacent to the target gene. The primers used for amplification of *ampD* deletion were FP 5′-GAGGCGGCATGTTAAAACTC-3′ and RP 5′-CCGAAAGAACGCTTCAAGAC-3′ (Table S1). For *ampE* deletion, primers employed were FP 5′-CGGGCCATTGTGATATTGCG-3′ and RP 5′-AGAGAAAACCGCCAAAGCCG-3′. To create *ampC* deletion primers used were FP 5′-GTTGTCACGCTGATTGGTG-3′ and RP 5′-CAGGCGCATAAATGTTTCC-3′ (Table S1). The PCR amplicons were electroporated into the recombinase-expressing cells and transformants were screened against kanamycin (25 mg/L) on LB agar plates. Deletions of *ampD* and *ampE* were verified by PCR using both cloning and deletion primers. Kanamycin cassette linked to FRT was cured after confirmation of deletion using the same procedure as described for *ampG* deletion (17 and data not shown). Kanamycin cassette was cured with the help of a plasmid, pCP20, expressing the flippase gene. A detailed description of the deletion method is provided in the Supplementary Materials (Figure S2).

### 3.3. Cloning of Predicted Beta-Lactamase-Inducing Genes

The genes implicated in the BLA induction pathway, *ampD* and *ampE*, were individually cloned in the pBAD18Kan vector. The clones were confirmed by double digestion of the recombinant constructs pKMD (*ampD* cloned in pBAD18Kan) and pKME (*ampE* cloned in pBAD18Kan) using the set of restriction enzymes employed for cloning (Figure S1).

### 3.4. Drug Susceptibility Testing (DST)

The micro-broth dilution method [39] was used to determine the minimum inhibitory concentration (MIC) of a selection of antibiotics against *E. coli* strains. The antibiotics used include ampicillin, amoxicillin, penicillin G, cefalothin, cefoxitin and oxacillin. The antibiotic concentrations were varied from 0.5 to 0.0005 mg/L and an inoculum of 105 cells was added in each well of the 96-well micro-titer plate. After incubation at 37 °C for 18 h, the bacterial growth was measured by determining the optical density of the culture at A600 nm using Multiskan Spectrum Spectrophotometer (MSS-model 1500; Thermo Scientific, Nyon, Switzerland). The MIC values for each antibiotic were determined by comparing the cell densities with control wells (without antibiotic) and results were interpreted as per CLSI guidelines. Experiments were carried out in three biological replicates for validation. The difference between MIC values of tested beta-lactam antibiotics against the wild type and Δ*ampD* and Δ*ampE* mutants were calculated to assess the intrinsic ability of the *E. coli* strain to resist beta-lactam antibiotics. Similarly, the susceptibility pattern against tested beta-lactams was checked for characterization of beta-lactamases used in this study.

### 3.5. Nitrocefin Hydrolysis Assay

The expression of BLAs under the presence of sub-inhibitory (1/8th of MIC) concentrations of beta-lactam inducers was assessed by nitrocefin hydrolysis. Nitrocefin is a chromogenic cephalosporin substrate that changes color from yellow to red which is directly proportional to the amount of BLA present. BLA genes harbored in *E. coli* cells were induced by sub-inhibitory concentrations of different beta-lactams and cells were grown till A600 nm reached about ~1.0. The cells were harvested and resuspended in 10 mM Tris-Cl buffer, followed by lysis using sonication. The expressed BLAs in the cell lysates were then assessed using 100 µM nitrocefin as reporter substrate at A492 nm. The value of the control cell (0.1 units) devoid of any BLA was subtracted from the readings obtained from samples.

## 4. Conclusions

Relying upon the observed changes in beta-lactam susceptibilities and altered expression of serine beta-lactamases (*viz*., CTX-M-15, TEM-1 and OXA-2) in absence of the AmpC beta-lactamase induction genes, *ampD* and *ampE*, the role of peptidoglycan recycling genes in the induction of serine beta-lactamase (other than AmpC) may be proposed. The role of AmpD amidase is to moderately facilitate a varying level of serine beta-lactamase expression where the absence of AmpD amidase enhances resistance in the strains producing TEM-1 beta-lactamase, while for the cells expressing CTX-M-15 and OXA-2, the effects are opposite. On the other hand, AmpE transporter might serve as a negative regulator for the expression of serine beta-lactamase.

## Figures and Tables

**Table 1:** Comparative beta-lactam sensitivities of wild type BW and single mutants of beta-lactamase induction genes—Δ*ampD*, Δ*ampE* and Δ*ampG*.

Antibiotics	MIC (mg/L)						
	BW	Δ*ampD*	Δ*ampE*	Δ*ampG*	Δ*ampDC*	Δ*ampCE*	Δ*ampGC*
Ampicillin	4	4	4	2	4	8	4
Amoxicillin	8	4	4	2	4	8	4
Cefalothin	8	4	4	2	8	16	8
Cefoxitin	8	8	4	4	2	4	1

**Table 2:** Beta-lactamase expression established by their beta-lactam resistance pattern in wild type *E. coli* (BW).

Beta-Lactamases	Antibiotics	MIC (mg/L)	
		BW	BW/BLA
CTX-M-15	Ampicillin	4	62.5
	Amoxicillin	8	125
	Cefotaxime	0.08	2.5
TEM-1	Amoxicillin	8	62.5
	Penicillin G	16	32
OXA-2	Amoxicillin	8	125
	Ampicillin	4	62
	Oxacillin	62	250

**Table 3:** Strains and plasmids used in this study.

Strain	Relevant Characteristic(s)	Remarks
XL1-Blue	F’*::Tn10 proA + B + lacIq* Δ*(lacZ)M15/recA1 endA1* *gyrA96* (NalR) *thi hsdR17 (rK–mK+) glnV44 relA1* *lac*	Stratagene, La Jolla, CA, USA
BW25113	F-::Δ*(araD-araB)567*, Δ*lacZ4787*(::rrnB-3), *λ-*, *rph-1*, Δ*(rhaD-rhaB)568*, *hsdR514*	The Coli Genetic Stock Center (CGSC)
Δ*ampC*	*ampC* deleted from BW25113	This work
Δ*ampD*	*ampD* deleted from BW25113	This work
Δ*ampE*	*ampE* deleted from BW25113	This work
Δ*ampG*	*ampG* deleted from BW25113	This work
Δ*ampGampC*	*ampG* and *ampC* deleted from BW25113	This work
Δ*ampDampC*	*ampD* and *ampC* deleted from BW25113	This work
Δ*ampEampC*	*ampE* and *ampC* deleted from BW25113	This work
**Plasmid**	**Relevant characteristic(s)**	
pGEM-T Easy	PCR cloning vector	Promega Corp., Madison, WI, USA
pKD46	Recombinase gene expressed by PBAD promoter, Amp^r^	CGSC
pCP20	Flippase gene cloned, Cam^r^	CGSC
pBM15	*bla_CTX-M15_* from NGM9 cloned in pBAD18-Cam	This work
pBT1	*bla_TEM-1_* from U-84 cloned in pBAD18-Cam	This work
pBO2	*bla_OXA-2_* from W-12 cloned in pBAD18-Cam	This work
pKMD	*ampD* gene from BW25113 cloned in pBAD18-Kan	This work
pKME	*ampE* gene from BW25113 cloned in pBAD18-Kan	This work
pKMG	*ampG* gene from BW25113 cloned in pBAD18-Kan	This work

**Table 4:** Antibiotic sensitivity of *E. coli* expressing serine beta-lactamases affected by *ampD*.

Beta-Lactamases	Antibiotics	MIC (mg/L)		
		Δ*ampD*	Δ*ampD*/BLA	Δ*ampD*/pKMD/BLA
CTX-M-15	Ampicillin	4	125	250
	Amoxicillin	4	32	62.5
	Cefalothin	4	32	125
TEM-1	Ampicillin	4	32	8
	Amoxicillin	4	125	16
	Cefoxitin	4	4	4
OXA-2	Ampicillin	4	8	500
	Amoxicillin	4	32	500
	Oxacillin	250	62	>500

**Table 5:** Effect of AmpE on beta-lactamase expression ascertained by altered beta-lactam sensitivity.

Beta-Lactamases	Antibiotics	MIC (mg/L)		
		Δ*ampE*	Δ*ampE*/BLA	Δ*ampE*/pKME/BLA
CTX-M-15	Amoxicillin	4	62.5	62.5
	Ampicillin	4	62.5	31.25
	Cefotaxime	0.1	1.6	0.4
TEM-1	Amoxicillin	4	125	4
	Ampicillin	4	125	2
	Cefoxitin	4	4	4
OXA-2	Amoxicillin	4	62.5	62.5
	Cefoxitin	4	4	2
	Oxacillin	250	500	250

## Data Availability

The data presented in this study are available in supplementary material.

## Supplementary Materials

The following are available online at https://www.mdpi.com/article/10.3390/antibiotics11010067/s1, Figure S1: Agarose gel electrophoresis for confirmation of clone construction, Figure S2: Gene deletion strategy using PCR products., Table S1: Sequences of primers used for deletion, Table S2. Changes in the beta-lactam sensitivity in presence and absence of AmpD Amidase, Table S3. Alteration in amoxicillin sensitivity of *E. coli* with respect to *ampE* gene.
