# Supplementary material for: Role of AmpC-Inducing Genes in Modulating Other Serine Beta-Lactamases in Escherichia coli
Source: Antibiotics (Basel). 2022 Jan 6;11(1):67. doi: 10.3390/antibiotics11010067 (PMC8772759; doi:10.3390/antibiotics11010067)
Supplement: Supplementary file 1 [file antibiotics-11-00067-s001.zip › antibiotics-1494485-supplementary.pdf]

Supplementary Material

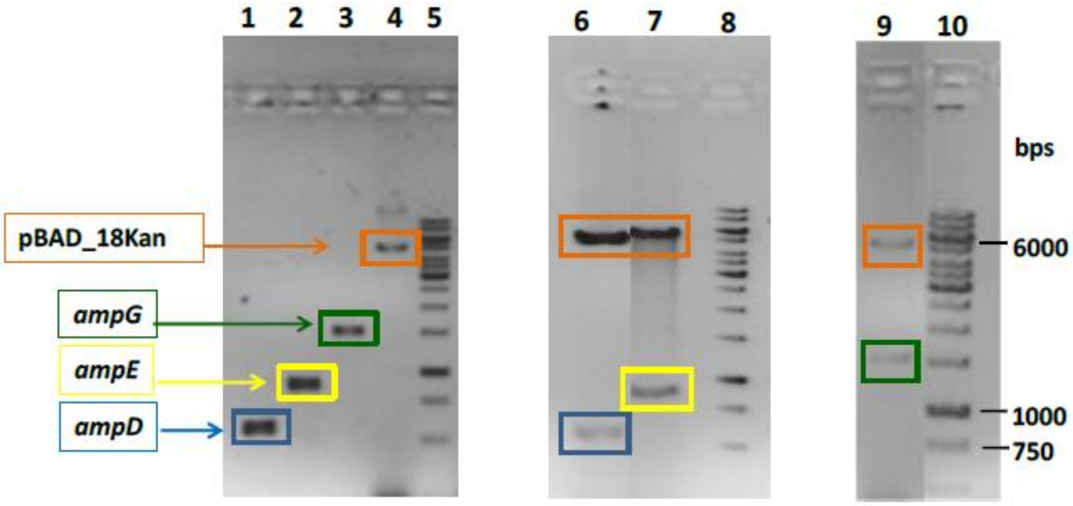

Figure S1: Agarose gel electrophoresis for confirmation of clone construction. Lane 1 – amplicon of *ampD*; lane 2 – amplicon of *ampE*; lane 3 – amplicon of *ampG*; lane 4 – digested pBAD_Kan vector; lane 5 – 1 kb DNA ladder; lane 6 – double digested construct pKMD (*Nhe*I & *Sac*I) exhibiting vector and insert *ampD*; lane 7 - double digested construct pKME (*Nhe*I & *Sac*I) exhibiting vector and insert *ampE*; lane 8 - 1 kb DNA ladder; lane 9 - 1 kb DNA ladder; and lane 10 - double digested construct pKMG (*Nhe*I & *Sac*I) exhibiting vector and insert *ampG*.

Method of gene deletion:

Deletion using PCR product: The strategy devised by Datsenko and Wanner was employed (Datsenko and Wanner, 2000). The kanamycin cassette (kan^r) was amplified along with FRT (flippase recognition target) sites on either side such that the amplicon is flanked on both sides by portions of sequences adjacent to the gene which is to be deleted.

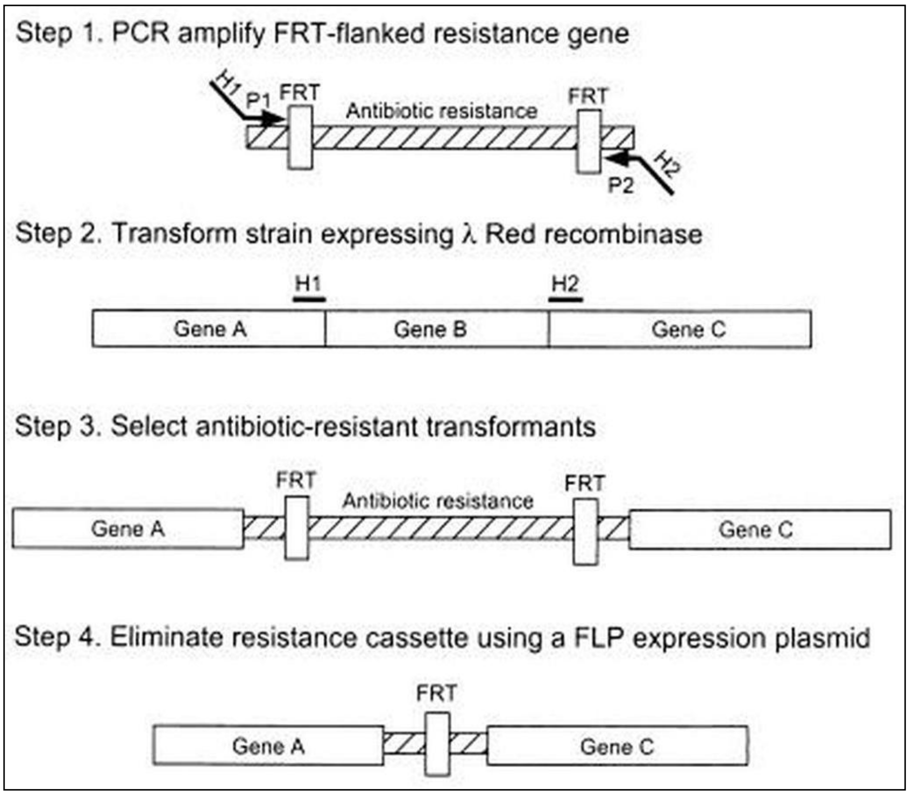

Figure S2: Gene deletion strategy using PCR products (Datsenko and Wanner, 2000)

Based on this, the following primers were employed to move deletions from one *E. coli* strain to the other.

Table S1: Sequences of primers used for deletion

Deletion primers	Primer Sequences	Product size (bps)
BW-ampD	FP 5'- GAGGCGGCATGTTAAAACTC – 3'<br>RP 5'- CCGAAAGAACGCTTCAAGAC – 3'	1500
BW-ampE	FP 5'- CGGGCCATTGTGATATTGCG -3'<br>RP 5'- AGAGAAAACCGCCAAAGCCG -3'	1500

The strain (from which gene was deleted) was transformed with a recombinase expressing plasmid pKD46 under the control of an arabinose promoter. This plasmid has an ampicillin marker and carries a temperature-sensitive origin of replication. The temperature sensitive plasmid pKD46 was transformed in the strain targeted for gene deletion (marker: ampicillin 100µg/mL; growth temperature: 30°C). This plasmid carries the recombinase gene induced by arabinose. The recombinase was induced in strain/pKD46 with 0.2% arabinose and competent cells prepared after recombinase expression. The PCR amplicons were electroporated into the recombinase expressing cells, and transformants were selected on LB agar plates with kanamycin at a concentration of 25mg/L. The deletion in transformants was verified by PCR using both cloning and deletion primers. On confirmation of deletion, a stock was prepared before proceeding for curing.

Curing of the kan^r cassette

Another temperature sensitive plasmid pCP20 was used to cure the kan^r cassette from the deletion mutants. The plasmid pCP20 was transformed into the deletion mutant and selected on chloramphenicol (20 mg/L) plates. The transformants were streaked on LB plates devoid of antibiotic and incubated at 37°C for the flippase expression. This step ensures curing of both the Kan^r cassette as well as the temperature sensitive plasmid pCP20 from the host strain. On the following day, the culture was re-streaked on two separate plates- one plate had kanamycin (25 mg/L) to check whether curing was successful and the other contained chloramphenicol (20 mg/L) for checking whether pCP20 was still present.

Table S2. Changes in the beta-lactam sensitivity in presence and absence of AmpD Amidase

**Antibiotics**	**MIC (mg/L)**		
	**BW**	**Δ*ampD***	**Δ*ampD*/pKMD**
Ampicillin	4	4	16
Amoxicillin	8	4	8
Cefalothin	8	4	8
Cefoxitin	8	8	8

Table S3. Alteration in amoxicillin sensitivity of *E. coli* with respect to *ampE* gene

**Antibiotics**	**MIC (mg/L)**		
	**BW**	**Δ*ampE***	**Δ*ampE*/pKME**
Amoxicillin	8	4	4
